# The effect of transcranial direct current stimulation on the interplay between executive control, behavioral variability and mind wandering: A registered report

**DOI:** 10.1016/j.ynirp.2022.100109

**Published:** 2022-06-11

**Authors:** Andreas Alexandersen, Gábor Csifcsák, Josephine Groot, Matthias Mittner

**Affiliations:** Institute for Psychology, The Arctic University of Norway (UiT), Norway

**Keywords:** Transcranial direct current stimulation, Mind wandering, Executive control, Non-invasive brain stimulation, Pupillometry, Electroencephalogram

## Abstract

Mind wandering (MW) is a mental phenomenon humans experience daily. Yet, we lack a complete understanding of the neural basis of this pervasive mental state. Over the past decade there has been an increase in publications using transcranial direct current stimulation (tDCS) to modulate the propensity to mind wander, but findings are diverse, and a satisfactory conclusion is missing. Recently, Boayue et al. (2020) reported successful reduction of mind wandering using high-definition tDCS (HD-tDCS) over the dorsolateral prefrontal cortex, providing preliminary evidence for the efficacy of HD-tDCS in interfering with mind wandering. The current study is a high-powered, pre-registered direct replication attempt of the effect found by Boayue et al. (2020). In addition, we investigated whether the effects of HD-tDCS on mind wandering would be prolonged and assessed the underlying processes of mind wandering using electroencephalography (EEG) and pupillometry during a finger-tapping random sequence generation task that requires the use of executive resources. We failed to find any evidence of the original effect of reduced MW during and after stimulation. When combining our data with the data from Boayue et al. (2020), the original effect of reduced MW caused by HD-tDCS disappeared. In addition, we observed increased occipital alpha power as task duration increased and increased midfrontal theta power preceding response patterns signaling high executive function use. Finally, tonic and phasic pupil size decreased as task duration increased yet, phasic responses were increased, while tonic responses were reduced preceding reports of MW. Additionally phasic pupil size also showed a tendency to be increased during periods of high executive function use. Importantly, none of the EEG or pupil measures were modulated by HD-tDCS. We conclude that HD-tDCS over the dorsolateral prefrontal cortex does not affect MW propensity and its neural signatures. Furthermore, we recommend that previously reported effects of tDCS on mind wandering and other cognitive functions should only be accepted after a successful pre-registered replication.

## 1 Introduction

Thoughts unrelated to the immediate here and now are often referred to as task unrelated thoughts (TUT) or mind wandering (MW; Antrobus et al., 1970). Being occupied with mind wandering is very common, both in everyday life (Killingsworth and Gilbert, 2010) and in experimental tasks (Smallwood and Schooler, 2006). In fact, research has found that humans spend up to half their waking hours occupied in thoughts unrelated to the here and now (Klinger and Cox, 1987). Researchers have argued that this phenomenon comes with both costs and benefits. Ruminating over past episodes is one of the major contributors to negative emotional states associated with mood disorders, such as depression and anxiety (Ottaviani and Couyoumdjian, 2013). Shifting our attention away from external stimuli can cause reduced performance in tasks that require focus, such as reading or driving (Yanko and Spalek, 2013). However, MW has been proposed to be beneficial in problem solving, future planning or tasks that require creativity (Pachai et al., 2016; Schooler et al., 2014; Smallwood and Schooler, 2015).

As to how mind wandering occurs, there is an ongoing dispute in the scientific literature. There is a consensus that executive functions (EF) are related to mind wandering, though there are different views on the nature of this relationship. The “executive function use” (EFu) view claims that mind wandering shares the same EF resources with ongoing tasks, and therefore, the individual must actively choose to allocate resources to either MW or the ongoing task (Smallwood and Schooler, 2015; Watkins, 2008). The “executive function failure” (EFf) view, on the other hand, states that MW is the result of the failure of the executive system to keep us focused on the primary task (McVay and Kane, 2010). Both the EFf and EFu accounts explain why performance on cognitive tasks decreases with the onset of MW. However, the distinction between the two is reflected in opposite predictions when one aims at modulating MW propensity. For example, EFf predicts that cognitive training targeting executive control mechanisms should be effective for attenuating increased prevalence of off-task thoughts in ADHD as patients get better at suppressing intruding thoughts, while such interventions are predicted to leave MW frequency unchanged or even increased in the EFu framework because an increase in EF can leave more capacity for MW. On the other hand, enhancing executive control might interfere with solving complex problems and creativity in the EFf, but not in the EFu view. In order to shed light on the role of EF in the onset of MW episodes, several cognitive tasks relying on EF have been developed to investigate mind wandering, such as the sustained attention response task (SART; Axelrod et al., 2015; Boayue et al., 2019; McVay and Kane, 2009) and the finger tapping random sequence generation task (FT-RSGT; Boayue et al., 2020). While the SART has been widely accepted as a task to investigate EF, a few problems have been highlighted recently: The task is very monotonous, and little EF is needed because target stimuli, to which prepotent response tendencies have to be inhibited, occur rarely (Boayue et al., 2020).

The FT-RSGT is a combination of a modified version of the classical random number generation task (Baddeley et al., 1998; Towse, 1998) and a finger-tapping task (Kucyi et al., 2017; Seli et al., 2013): It consists of a combination of rhythmic finger-tapping in response to an ongoing metronome and the generation of random sequences by pressing the two available response-buttons in a random sequence. The idea behind this task is as follows: Generating random sequences is a task that draws heavily on executive resources. Consequently, we expect the randomness of the generated sequence to be related to the amount of executive resources diverted to it. In the context of mind wandering, this has been confirmed by the finding that sequences generated while engaged in MW are typically less random (Boayue et al., 2020; Teasdale et al., 1995). Furthermore, behavioral variability (BV) as measured by the deviation of the taps from the on-going metronome in finger-tapping studies has also been found to be an indicator of mind wandering (Kucyi et al., 2017; Seli et al., 2013), with behavior becoming more variable when attention is drawn away from the task. Since the metronome is fast-paced, the FT-RSGT provides good temporal resolution on both EF (changes in sequence randomness), and behavioral variability. By combining both measurements in a single experiment, the dynamic interplay between behavioral variability and executive function can be investigated. A previous study employing the FT-RSGT found that the two measures interact in relation to self-reported mind wandering: When randomness was high and EF was strongly engaged, there was a strong relationship between BV and MW, but this relationship was weakened when less executive resources were diverted to the task (Boayue et al., 2020). This intriguing result may speak to the non-unitary nature of MW and may help in distinguishing different states underlying MW (Allan Cheyne et al., 2009; Mittner et al., 2016).

In addition to studies elucidating the nature of MW on a behavioral level using cognitive tasks, there has been an increase in research focusing on the role of neural networks in MW (Andrews-Hanna et al., 2010; Dixon et al., 2018; Fox et al., 2013), and in methods for manipulating their activity with non-invasive brain stimulation (NIBS) techniques (Axelrod et al., 2015, 2018; Boayue et al., 2019; Coulborn et al., 2020; Csifcsak et al., 2019; Csifcsák et al., 2018; Filmer et al., 2021). To identify these networks, researchers have attempted to locate neural markers of mind wandering, such as electrophysiological signatures recorded by electroencephalography (EEG; Braboszcz and Delorme, 2011; Kawashima and Kumano, 2017), functional magnetic resonance imaging (fMRI; Chou et al., 2017; Christoff et al., 2009; Mittner et al., 2014) or both (Groot et al., 2021). For example, an EEG study found reduced mismatch negativity (MMN) event-related potentials during MW, possibly indicating attenuated monitoring of the sensory environment and weaker responses to unexpected events (Braboszcz and Delorme, 2011). Additionally, alpha oscillations have been observed to increase during wakeful rest and are thought to mediate attentional lapses and task unrelated thought (Braboszcz and Delorme, 2011; Macdonald et al., 2011; O'Connell et al., 2009). In particular, it has been hypothesized that increased alpha power reflects a top-down control measure to prevent brain regions involved in competing processes to interfere with task performance (Jensen et al., 2012; Sauseng et al., 2005).

In addition, measuring changes in pupil diameter (pupillometry) is yet another tool that is increasingly being used to get a better understanding of how and when mind wandering occurs (Mittner et al., 2016; Schwalm and Jubal, 2017). Evidence suggests that tonic and phasic pupillary responses are modulated by norepinephrinergic signalling (Joshi et al., 2016), and that phasic pupillary responses are correlated to mental effort (van der Wel and van Steenbergen, 2018). This makes pupillometry a useful tool to investigate neural processes underlying MW, which has been hypothesized to be driven by the norepinephrinergic system (Mittner et al., 2016). Another advantage of pupillometry is that it can easily be combined with other measurements such as EEG or fMRI in MW research, without causing interference or complicating the signals (e.g., Groot et al., 2021). In addition, pupillary responses are elicited by stimuli in both the visual (Barbur et al., 1992) and auditory domains (Zekveld et al., 2018) and can therefore be studied across different modalities. By combining multiple measures with high temporal resolutions such as EEG and pupillometry, with cognitive tasks designed to induce MW, it is possible to get a better understanding of the spatiotemporal relationship of MW and their neural correlates (Groot et al., 2021). Using these tools, researchers have observed the involvement of several networks working together to produce and maintain the phenomenon of MW (Christoff et al., 2016; Groot et al., 2021; Mittner et al., 2014; Poerio et al., 2017).

Evidence suggests that activity in brain regions of the default mode network (DMN) is anti-correlated with activity in networks underlying the processing of task-relevant external stimuli (Andrews-Hanna et al., 2010; Smallwood et al., 2012a). It has been proposed that the DMN is responsible for providing the content to which we can mind wander by retrieving traces from episodic memory (Kam et al., 2022, Andrews-Hanna et al., 2010; Dixon et al., 2018; Fox et al., 2013, 2015). However, the DMN alone is not responsible for the experience of mind wandering, and spontaneous thoughts most likely depend on the complex interplay between multiple different networks (Kam et al., 2022; Christoff et al., 2016; Fox et al., 2015). The frontoparietal control network (FPCN) along with the dorsal attention network (DAN) and the ventral attention network have been observed to regulate when and where we shift our attention (Christoff et al., 2016; Smallwood et al., 2012a). During cognitive tasks, activity is modulated in the FPCN along with the DAN, which seems to be associated with attentional shifts between task demands and MW (Brissenden et al., 2016; Christoff et al., 2016). Therefore, a cooperation between systems involving control and coordination, such as the FPCN, coupled together with the DMN for content, might be involved in the dynamic interplay in how humans select, evaluate and guide which content is available for conscious thought (Andrews-Hanna et al., 2010; Christoff et al., 2016; Fox et al., 2015, 2015, 2015; Smallwood et al., 2012b; Yeo et al., 2011). The importance of executive control in the MW process makes the FPCN an excellent target for interfering with mind wandering using NIBS methods.

The dorsolateral prefrontal cortex (DLPFC) is one of the core regions of the FPCN and has frequently been targeted using brain stimulation due to its superficial location and hence, easy accessibility for NIBS. Perhaps, the most widely applied NIBS method is transcranial direct current stimulation (tDCS), which operates by applying low-intensity direct currents to the head to induce an electric field across the cortex (Stagg and Nitsche, 2011). Depending on the polarity of the stimulation, this electric field can either depolarize or hyperpolarize the neuronal resting potential of cortical pyramidal neurons, making it a versatile tool for modulating brain activity. Some studies provide evidence for tDCS to induce neuroplastic effects when applied for longer stimulation periods (Lefaucheur et al., 2014, Nitsche and Paulus, 2001), making it plausible that tDCS can be used to create lasting effects beyond the stimulation period.

Initially, several studies reporting successful modulation of mind wandering propensity using traditional bipolar tDCS over the DLPFC provided an optimistic outlook (Axelrod et al., 2015; Kajimura et al., 2016; Kajimura and Nomura, 2015). However, several subsequent studies have failed to replicate this effect (Coulborn et al., 2020), including a large-scale, pre-registered direct replication study (Boayue et al., 2019). This suggests that the initial positive results that were based on very low sample-sizes might have been overly optimistic (but see Axelrod et al., 2018; Csifcsak et al., 2019). Discrepancies between study findings might stem from the weak focality of traditional bipolar tDCS montages (Csifcsák et al., 2018), which, combined with the considerable variability in stimulation protocols and cognitive tasks across MW studies increase the likelihood to find inconsistent results. While newer registered reports (e.g., Filmer et al., 2019, 2021) show promising results in increasing MW with tDCS, it seems to be even more attractive to be able to reduce MW during a demanding task from an application point of view.

The results regarding a reduction of MW when employing tDCS are also mixed. Kajimura and colleagues were the first to publish preliminary evidence of successfully reducing TUTs when anodally stimulating the right inferior parietal lobule (return electrode over left DLPFC) using a traditional bipolar tDCS montage (Kajimura et al., 2016; Kajimura and Nomura, 2015). However, a pre-registered study with a higher sample size employing identical tDCS-protocols found the opposite effect (Filmer et al., 2021), mirroring the pattern of non-replicable effects for the studies aiming to increase MW with non-focal tDCS protocols reviewed above. A recent study implementing a more focal high-definition tDCS (HD-tDCS) protocol showed promising results in reducing the amount of mind wandering observed during the FT-RSGT (Boayue et al., 2020). Unfortunately, the final analysis pipeline from this study was not pre-registered, and therefore provided only preliminary evidence for the found effect. In the current study, we aim to replicate the effect found by Boayue et al. (2020) using a pre-registered, high-powered study design and a registered report publication format, involving stage-1 peer-review of the protocol and analysis plan. Based on the outcomes of that previous study, we formulated and pre-registered four hypotheses: (1) We expect behavioral variability (BV) to be increased prior to self-reports of mind wandering when compared to on-task periods, (2) we expect the utilization of executive resources (operationalized by approximate entropy, AE, of the generated tap-sequences) to be reduced prior to mind wandering, (3) we expect an interaction effect of BV and AE such that the positive correlation between BV and MW is more pronounced during periods of high AE, and (4) we expect the propensity to mind wander to be reduced in the real relative to the sham stimulation group during the stimulation block of the experiment (online effect of HD-tDCS).

In addition, in a more exploratory approach, we extend the previous study in an attempt to gain a better understanding of the neural mechanisms underlying the pre-registered effects. These additions comprise (1) extending the original protocol with a third (offline) experimental block that enables assessing whether the effects of HD-tDCS outlast the duration of stimulation, (2) collecting pupillometry data to investigate how phasic and tonic responses are changed in periods preceding on-task vs. MW reports, and recording EEG to assess if neurophysiological measures of (3) sensory prediction errors (the mismatch negativity), (4) executive control (frontal midline theta oscillations) and (5) attention (posterior alpha activity) are influenced by mental state (on-task vs. MW), HD-tDCS (real vs. sham) and their interaction. These additional goals are secondary to the replication attempt, specified more loosely (i.e., not pre-registered in detail) and do not interfere with the exact replication protocol.

## 2 Methods

All materials, simulations and analyses are available in a public repository hosted by the Open Science Framework (OSF) at https://osf.io/cv24f/. The repository was registered (frozen) before data collection such that none of the materials can be covertly changed after data has been collected. At the time of pre-registration, data from 43 participants had already been collected, yet no data were accessed until data collection was completed. In addition, the full analysis was pre-registered at the OSF platform ahead of collecting the first dataset (registration document available at https://osf.io/9ytgp). The data were stored without reference to the stimulation condition to enable a blinded analysis (see section 2.2.3).

### 2.1 Participants

Participants were selected and recruited randomly amongst healthy adults in Tromsø through advertisements and social networks. Ethical approval was approved by the institutional ethics committee of the Department of Psychology at the UiT - The Arctic University of Norway. Participants received gift-cards (worth 300 NOK, approximately 30 EUR), or course credits as compensation for taking part in the study. In accordance with the sample size calculations (see section 2.8.2), 100 participants were recruited (50 valid datasets in each experimental group). In case a participant failed to provide a complete behavioral dataset, the dataset was excluded, and the participant replaced with a new one. Participants were replaced if the task was not completed, behavioral data were incomplete due to technical issues, stimulation equipment failed, or similar technical issues which rendered the behavioral dataset incomplete. The participant was not replaced if the EEG or pupillometry measurement was contaminated or failed. The inclusion criteria were: Fluent in either English or Norwegian, signed consent-form, aged between 18 and 35 years, no psychiatric/neurological conditions (e.g., depression bipolar disorder, epilepsy etc.) currently or in the past, not under the influence of psychotropic drugs (except caffeine and nicotine), not taking central nervous system medications (e.g., antidepressants, antiepileptic drugs), good or corrected eyesight (to read the instructions on the screen), not extremely tired or hungry on the test day. All these criteria were assessed by self-report on the day of the experiment ahead of the experiment. After recruitment, participants were randomly allocated to either a sham or anodal stimulation group by a randomization list (see section 2.2.3 for more information about blinding).

### 2.2 Design and procedure

#### 2.2.1 Lab, location, personnel

The experiment was conducted in a lab containing an isolated experimental chamber that ensures a disturbance-free environment and reduces artifacts from environmental sources in the EEG. During the experiment, the outer door to the experimental room was locked so that no one could enter the lab without permission. The participant was placed in the experimental chamber that allows to control sound and lighting conditions. During the completion of the experimental task on the computer, the experimenter waited outside the chamber and monitored progress. Before starting the experimental task, room illuminance was measured and noted down on the datasheet (https://osf.io/arzxe/), and the participant was equipped with a high-quality stereo headset (Multi-Function Headset210, Trust International B.V., Dordrecht, Netherlands) in front of a 19” flat-screen monitor. The distance to the monitor was standardized (70 cm) and the height of the entire table was individually adjusted by the experimenter to fit each participant. The experimental computer ran PsychoPy3 (release v2020.1.3) and was set up so no other disturbing background processes were running. Since the experiment was conducted during Covid-19, necessary precautions had to be made. A full list of contagion preventive measures can be found at https://osf.io/8ud5e/. Approval for these measures was obtained from the Faculty of Health Sciences at UiT - The Arctic University of Norway.

Data were acquired by four trained experimenters. All experimenters received the same standardized instructions for conducting the experiment and were required to practice on at least two pilot subjects before acquiring real data. Instructions to the participants were given in a written format to keep experimenter influences at a minimum, but there were opportunities for participants to ask questions or receive clarifications from the experimenter. All instructions are available in both Norwegian and English and can be found at https://osf.io/dxwqj/for English, and https://osf.io/5z6bx/for Norwegian.

#### 2.2.2 Design

**Fig. 1:**
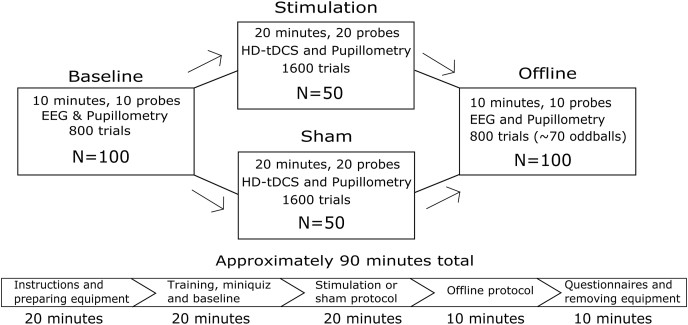
Flowchart of the experimental session. All participants started by completing an identical baseline block (800 trials). Next, they were randomized into two groups to complete the stimulation block (1600 trials) where they received either real or sham HD-tDCS. Finally, all participants completed an identical offline block (800 trials) that included oddball stimuli (∼70 oddballs).

The experiment was set up as a mixed design with one between-subject factor (Group: sham vs. real stimulation) and one within-subject factor (Block: baseline, stimulation, offline), see Fig. 1.

The main analysis was an ordinal regression analysis of mind-wandering propensity as a function of transformed AE (approximate entropy), BV (behavioral variability), their interaction, Trial, Block, Group, and the Group × Block interaction (details further down). The study implemented the exact same study protocol as our previous study (Boayue et al., 2020), up to the point where the stimulation block of the study was completed. The only divergence from the original study (Boayue et al., 2020) up to that point was that we also collected pupillometric and EEG data. After the stimulation part, the study deviated from the previous protocol by collecting data in an additional offline block, during which oddball stimuli were presented in addition to the ongoing default metronome sound (details below). The task, the FT-RSGT, was developed in the original study (Boayue et al., 2020), and the study script is available at https://osf.io/t2b3m/. The study was administered either in English or Norwegian language, depending on the preference of the participant. All experimental materials and experimenter instructions are shared under the folder “materials” at https://osf.io/cv24f/.

#### 2.2.3 Blinding

The experiment was conducted in a triple-blind manner: Neither the experimenter nor the participant nor the analyst knew which treatment group each participant was assigned to. This was achieved by creating a collection of 100 stimulation protocol files, one for each participant with a unique code. Fifty of these files contained instructions for the stimulator to apply active stimulation while the other 50 files applied sham stimulation. For the analysis, we created a list that contained the unique participant codes paired with arbitrary labels “A” and “B”, coded by investigator GC, to blind the main analyst MM, making it impossible to know which participant received sham or real stimulation during the main analysis. Only after the results were finalized was the identity of the groups revealed. The stimulation software (NIC2, version v2.0.11.1) on the stimulation computer allowed for a double-blind mode, where it was impossible for the experimenter to identify the stimulation protocol as the visual display on the stimulation computer was identical in both cases. It is a known issue that side-effects such as itching and redness of the skin under the electrode can reduce the efficacy of the blinding in tDCS studies with bipolar montages (Turi et al., 2019). To increase the efficacy of the blinding, an anesthetic cream containing 2.5% lidocaine and 2.5% prilocaine (EMLA) was applied under the stimulation electrodes prior to stimulation. After a minimum of 15 min allowing the cream to be absorbed by the skin, any remaining cream was removed through the holes of the EEG cap using a cotton swab. To test the efficacy of the blinding, participants were asked at the end of the experiment to report if they believed they received real stimulation versus sham by answering a questionnaire consisting of a Likert scale ranging from 1 to 7 where 1 was “definitely not stimulation, and 7 was “definitely stimulation”. The questionnaire can be found at https://osf.io/auf82/. We have not observed compromised blinding in our previous application of this stimulation protocol (Boayue et al., 2020).

### 2.3 HD-tDCS protocol

**Fig. 2:**
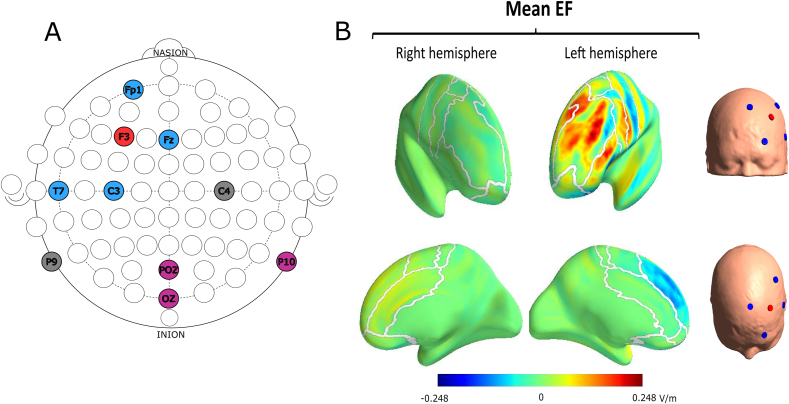
Positioning of electrodes (A) and electric field produced by the HD-tDCS (B). Electrode positions are according to the international 10/20 system (A). Anode (red) at F3, Cathodes (blue) at T7, C3, Fz and Fp1. Our equipment allowed us to place 3 more electrodes for EEG sampling (purple), which were placed at positions POz, Oz and P10, with the ground and reference electrodes (grey) placed at C4 and P9, respectively. Simulation of the normal component of the electric field induced by the 4x1 HD-tDCS montage (B), averaged over N = 18 individual datasets (realistic head models created from MR images of healthy adults), figure repurposed with permission from Csifcsák et al. (2018). (For interpretation of the references to colour in this figure legend, the reader is referred to the Web version of this article.)

The stimulation was delivered using a 4x1 HD-tDCS montage using a Starstim Neckbox (Starstim tCS, NE Neuroelectrics), and PISTIM EEG & tES Ag/AgCl electrodes (diameter: 12 mm). In this montage, one anode is surrounded by four cathodes (Fig. 2). This setup has been previously shown to create a strong electric field in and around the targeted brain region (Csifcsák et al., 2018, Fig. 2). In the real stimulation condition, participants received continuous stimulation at 2 mA intensity for 20 min, with 30 s fade-in and 30 s fade-out periods. The sham protocol applied the fade-in and fade-out periods at the start of the stimulation block and then produced no currents until a final fade-in and fade-out period at the end of the block.

Positioning of the electrodes was measured with a positioning cap, in accordance with the international 10/20 system. Anode intensity was set to 2 mA and returned evenly at each of the cathodes (Fig. 2). Instructions on how to fit the cap were standardized and practiced by all experimenters. Head circumference was measured to determine size of the cap (M = 54 cm, L = 57 cm, XL = 62 cm). Once the correct cap was fitted to the participant's head, symmetry was checked by measuring the distance between inion, nasion, preauricular point and the respective electrode positions. The full procedure can be found at https://osf.io/u8n7x/.

### 2.4 The cognitive task

In order to establish a comparable level of understanding of the meaning of “randomness” when applied to a sequence of button-presses, participants were presented a written instruction using the flipping of a coin as an example. They were told that their button-presses should resemble the result of repeatedly flipping a fair coin and that, therefore, each of the two buttons should have equal probability of being pressed in each trial (see instructions at https://osf.io/pr2m9/). The participants then received instructions on the FT-RSGT through the experimental software and completed a short training session (60 s). After the training session the participants were asked to fill out a mini-quiz where they had to answer seven simple questions that were designed to check whether they understood the instructions regarding randomness, the task instructions and how to answer the thought probes. Wrong answers were corrected and, if necessary, the participant received an additional explanation. The mini quiz is available here at https://osf.io/c7ajk/.

During the task, participants were instructed to press two buttons with their left or right index finger in a random order. Participants were also instructed to match every single button press as accurately as possible to the occurrence of a rhythmic tone. The tones had a pitch of 440 Hz and were presented for a duration of 75 ms, with an inter-stimulus interval of 750 ms via high-quality stereo headphones.

The stimulus (the rhythmic tone) was presented 800 times during the baseline block, 1600 times during the stimulation block and 800 times during the offline block. In the third, offline block, approximately every 10th (8–12) stimulus had a higher pitch (880 Hz), but never within the first 10 stimuli after block start, or in the first 10 stimuli after a thought probe. These oddball stimuli were used for analyzing the mismatch negativity (MMN) in the EEG. The participants were interrupted regularly by experience sampling thought probes, asking where the participants focus had been prior to the probe. The thought probe consisted of a Likert scale ranging from 1 to 4, with 1 labeled “clearly ON TASK” to 4 being “clearly OFF TASK”. The exact question asked in the probes was “Where was your attention (your thoughts) directed right before this question appeared?”. The number of thought probes was fixed to 10 probes in the baseline and offline blocks, and 20 probes in the online stimulation block. Probes appeared at a random interval between 40 and 80 s (uniformly selected).

### 2.5 Behavioral measurements

#### 2.5.1 Behavioral variability

Behavioral variability was measured as the standard deviation of the inter-tap-intervals using the last 25 taps preceding a thought probe, and this index was z-transformed across subjects. No responses or trials were excluded during the calculation of the BV measure. That way, both missing responses as well as double-taps acted towards increasing the measure. This procedure is identical to the one used in Boayue et al. (2020).

#### 2.5.2 Approximate entropy

To measure randomness in the sequences the participants created, we used a statistic called approximate entropy (AE; Pincus, 1991) which is defined at sequence level. AE is a measure for evaluating the irregularity in any given sequence and is parameterized by parameter *m*. AE(*m*) measures the logarithmic frequency with which blocks of length *m* that are close together, remain close together for blocks augmented by one position (Pincus and Singer, 1996; Pincus and Kalman, 1997). The higher the AE(*m*) score, the more irregular the sequence. In a previous study, Boayue et al. (2020) determined the optimal parameter for subsequence length to be *m* = 2 and we have used the same value for our analyses. AE was calculated on the same subsequences of the 25 taps preceding each thought-probe and was transformed using the formula $AE_{trans}=-\log(\log\left(2\right)-AE_{raw})$.

### 2.6 Additional measurements^1^

#### 2.6.1 Pupillometry measurement and preprocessing

During stimulus presentation, pupil size was recorded at a rate of 500 Hz from both eyes with a desktop-mounted infrared video-based eye tracker (Eyelink 1000, SR Research), connected to a laptop running EyeLink Portable Duo (v6.12) software on Linux. Before the task started, the experimenter calibrated the equipment for pupillometric data collection. The eye tracker, chin rest and monitor were in a fixed position (distance between eyes and tracker/monitor), with the only adjustment being the height of the desk which is adjustable with an electric motor to ensure a comfortable position for our participants.

Preprocessing and analysis of the pupillometric data were conducted using the pypillometry package (Mittner, 2020) in Python. Raw pupillometric data from both eyes were imported and the pupil data from the eye with the least missing datapoints for each subject was selected for further processing. Next, blink-detection using the procedure detailed in Mathôt (2013) was applied. The velocity parameters of the blink-detection algorithm were optimized for each individual to ensure that individual transients associated with blinking were adequately spotted while attempting to minimize the detection of non-blink artifacts, a list of all the parameters for each participant can be found on OSF (https://osf.io/tqn6m/). This procedure relied on visually inspecting and evaluating the raw and preprocessed signals by an expert analyst (JMG). Next, blinks that were close together in time (<100 ms) were merged to avoid interpolation artifacts. After linearly interpolating blinks using the method described in Mathôt (2013), a 5 Hz lowpass-filter (Butterworth) was applied to the continuous data. In addition, full datasets were excluded from the pupillometric analyses when judged to be of insufficient quality for further analyses (see section *3.4.1 Missing data)*.

Even after following the rigorous preprocessing steps described above, 25 datasets for the baseline part, 30 datasets for the stimulation part, and 23 for the offline part had to be excluded due to extreme blinking, exceeding our criterion that more than 50% of data were missing, or technical issues with pupil tracking. There were 61 subjects (31 real, 30 sham) with complete data on all three parts. Trial-by-trial measures of baseline and pupil-response were extracted using two methods. First, we used a classical approach in which the mean pupil-diameter (PD) in a window preceding each metronome sound ([−200, 0] ms) were used as baseline while the response was quantified as the difference of mean PD in a time-window following the metronome sound ([500, 700] ms) and the baseline. Because of the fast-paced design of our experiment, pupillary responses to the events overlapped and showed an accumulation of the signal because of the slow pupillometric response-curve (Hoeks and Levelt, 1993). To compensate for this, we used a novel deconvolution-based estimation approach (Mittner, 2020) to estimate both baseline and pupillary responses per trial.

First, the data were downsampled to 250 Hz and high prominence troughs (negative peaks) were determined throughout the pupil signal. Tonic (baseline) fluctuations were estimated using B-spline basis-functions constrained to pass through these peaks in two iterations. After the first iteration, the tonic estimate was subtracted from the signal, followed by subtracting modeled pupil-response functions (PRF; Hoeks and Levelt, 1993) at known task events. This ensured that the second iteration of B-spline basis-functions (the final tonic estimate) remained below the actual pupil signal. Single-trial tonic pupil size features were calculated at every stimulus (metronome) onset. To model phasic pupil responses to task-related events, regressors for every stimulus and tap onset were convolved with the pupil-response function (PRF; *h* = *t*^*n*^*e*^*-n*^^/^^*tmax*^, where *n* = 10 and *t*_max_ = 900; Hoeks and Levelt, 1993) and fitted with a non-negative least-squares solver (Lawson and Hanson, 1995). To avoid multicollinearity between stimulus and tap onsets, the *b*-coefficients from all events within the [−200, 200] ms window around stimulus onset were summed up together and assigned to that trial. Single-trial tonic and phasic pupil responses were standardized (z-scored) within subjects.

#### 2.6.2 EEG recording and preprocessing

We measured the EEG during the baseline and offline blocks from all 8 electrodes (see Fig. 2). Since the 5 stimulation electrodes were fixed (for replication purposes), we decided to place the last 3 available electrodes at POz, Oz and P10, respectively. These 8 electrodes were used to collect data for analyzing the MMN event-related potential, occipital alpha and midfrontal theta power (see section 2.9.5).

To ensure low impedance, any remaining superfluous EMLA cream was removed, and the electrodes were administered with conductive gel (SIGNA) before they were fit into the cap. The impedance of the electrodes was kept under 10 kΩ. The EEG was collected at 500 Hz, without online frequency filters. Data were analyzed using BrainVision Analyzer 2 (BrainProducts GmbH, Gilching, Germany) and EEGLAB (Delorme and Makeig, 2004). Continuous EEG was filtered with a 4th-order zero phase-shift Butterworth filter (24 dB/oct) with a low cut-off of 0.5 Hz and a high cut-off of 30 Hz.

The MMN was analyzed at Fz, from data collected in the offline block only (since we presented oddball stimuli in that block exclusively). We started with re-referencing the data to the average signal recorded above the mastoids (P9 and P10) and extracting epochs relative to metronome sound onset ([−100 ms, 500] ms), separately for standard and oddball stimuli. Epochs containing artifacts associated with blinking, eye movements, muscle activity or other extracerebral sources were removed. The remaining epochs were baseline corrected ([−100, 0] ms) and averaged. For each participant, we calculated difference waveforms (oddball – standard) and created grand average waveforms using data available from all participants. We anticipated finding an event-related potential with negative polarity between [120, 300] ms post-stimulus.

To ensure that this peak corresponds to the MMN, we verified that the same component switched polarity at posterior-inferior electrodes (POz and Oz). Once identified, mean MMN amplitudes were measured at Fz (referenced to P9/P10) for each participant, in a 40 ms time-window centered at the MMN peak (120 ms) of the grand averaged difference waveform. Occipital alpha power was measured at electrodes POz and Oz from data collected in the baseline and offline blocks. The data were re-referenced to electrode Fz, and continuous EEG were divided into 1000 ms-long epochs and baseline corrected using the mean over the whole epoch. As the first step, we verified whether occipital alpha power was related to subjective reports of MW. For this purpose, we merged data from the two blocks, and separated epochs preceding on-task vs. off-task reports (25 epochs preceding each thought-probe). After removing epochs with artifacts, we applied a Fast Fourier Transform (FFT) with a Hanning window of 10%, resulting in a frequency resolution of 0.97 Hz. We extracted the sum of power values corresponding to the alpha frequency band (8–12 Hz) for each participant and compared alpha power between on-task vs. off-task reports. We hypothesized to find enhanced occipital alpha activity during periods of mind wandering relative to the EEG collected prior to on-task reports on a group level. Once the association between posterior alpha activity and MW was established, we proceeded with analyzing data from the baseline and offline blocks separately. However, to increase statistical power, we used all epochs, not just those preceding thought-probes: We extracted occipital alpha power and compared changes between the first and the last blocks, separately for the real vs. sham HD-tDCS groups.

Finally, we analyzed frontal midline theta power (MFT), which has been associated with the implementation of executive control in various cognitive tasks (Cavanagh and Frank, 2014). In this regard, we expected this neurophysiological marker to be related to the successful generation of random sequences during the FT-RSGT. We used data recorded at Fz, both during the baseline and the offline blocks. The EEG was re-referenced to the mean signal from electrodes P9 and P10, epochs centered around finger taps were extracted ([−1000, 1000] ms, allowing temporal overlap between subsequent epochs), and epochs containing artifacts were removed. In order to verify that frontal midline theta power is related to executive control and its behavioral correlate (AE), we merged data from both blocks, but separated epochs corresponding to high and low AE values (median split for each participant). Next, we performed a time-frequency decomposition of the segmented data using continuous complex Morelet wavelets (from 1 Hz to 30 Hz in 30 linear-spaced frequency steps, Morlet parameter c = 3), and averaged data corresponding to high-vs. low-AE epochs separately. Theta power was assessed in [−500, 0] ms (since we expected executive control to be implemented prior to motor responses), in the theta frequency range (4–8 Hz). We excluded trials where additional behavioral responses were observed in this time-window. Since we found the anticipated stronger theta power for high-AE epochs on the group level (including all participants), we applied the same analysis pipeline to extract theta power for both experimental blocks separately and extracted MFT power in the time-frequency window that was used in the merged analysis. This data was used to evaluate changes in frontal theta activity between blocks and groups.

#### 2.6.3 Feedback questionnaires

After the task was completed, the participants were asked to answer a questionnaire as well as a few questions asked by the experimenter. The written questionnaire consisted of six items asking about potential distraction from wearing gloves (as part of the preventive COVID-19 measures), discomfort from the electrodes, motivation, confidence, and intention of mind wandering as well as blinding efficacy. Items were rated on a 7-point Likert scale, and the full questionnaire can be found at https://osf.io/auf82/.

The verbal questions covered randomization strategy, mind-wandering content and an open feedback question for future improvements. These questions can be found at https://osf.io/7jxwa/.

### 2.7 Deviations from the original study

Even though we attempted a direct replication of Boayue et al. (2020), we acknowledge that there are subtle differences between the data collection protocols in the two studies. In the baseline block, participants in our study were already equipped with the EEG cap because we intended to record EEG data already during that block. In Boayue et al. (2020) on the other hand, there was no EEG recording, and thus the EEG cap was not placed on the participants’ head before the stimulation block. Furthermore, since the original study put on the cap and electrodes between the baseline and stimulation blocks, there might have been a small difference (between 1 and 3 min) in the duration between the baseline and stimulation blocks.

Another difference between the two studies is that the EMLA cream was removed with alcohol after taking off the EEG cap. In our study, we had to remove the superfluous EMLA cream through the holes in the EEG cap and the application of cleansing alcohol was therefore impractical. In addition, we recorded pupillometry during the experiment and therefore needed to calibrate the equipment before the start of the study. Finally, due the ongoing Covid-19 pandemic, both the experimenters and the participants wore protective equipment (masks, gloves, coats) though participants were allowed to remove the mask during the actual experimental task.

However, we believe that these deviations from the original protocol were negligible, and we do not see a theoretical reason that any of these changes should compromise our replication attempt.

### 2.8 Statistical methods

#### 2.8.1 Analytic methods

All analyses of behavioral data were implemented in the R programming language (R Core Team, 2015) and Stan (Carpenter et al., 2017), using the packages BayesFactor (Morey and Rouder, 2015), rstan (Stan Development Team, 2016) and brms (Bürkner, 2017). EEG data was analyzed in JASP (JASP Team, 2021), for preprocessing see section 2.6.2. Pupillometric preprocessing was done using the pypillometry package (Mittner, 2020) in Python v.3.4 (Van Rossum and Drake, 2009).

We used a single statistical model to test all four hypotheses. To circumvent some of the problems highlighted by Liddell & Kruschke (2018) when analyzing thought probe data as a continous rather than an ordinal variable, we used a Bayesian hierarchical ordered-probit regression model with random intercepts at the subject level and experimental block nested within subject (see Boayue et al., 2020). The model included BV (behavioral variability), AE (approximate entropy), their interaction, Trial (probe number), Group (sham vs. real stimulation), Block (baseline vs. stimulation) and the interaction between Group and Block as independent variables. The model also included random intercepts at the subject level nested within Block. The full model description in R-code (implemented using the package “brms”) can be found at https://osf.io/3fcsx/.

Based on this model, we calculated the posterior mean and 95% highest-density intervals (HDI) of the regression coefficients as well as evidence ratios (ER). Evidence ratios are calculated by dividing either the total positive or negative posterior mass by the total posterior mass on the other side. Specifically, the ER for a positive effect, $ER_{+}=\frac{P\left(x>0|\theta \right)}{P\left(x\leq 0|\theta \right)}$ and the ER in favor of a negative effect is the inverse $ER_{-}=\frac{P\left(x\leq 0|\theta \right)}{P\left(x>0|\theta \right)}$. These ratios can be interpreted as odds-ratios, e.g., how much more likely a positive effect is compared to a negative or no effect. Regarding our four main hypotheses, we calculated the area under the marginal posterior distribution of each parameter that is larger (for BV and AE × BV) or less than zero (for AE and Block × Group). If this area was larger than 0.95, we concluded that our hypothesis was supported. In case the calculated statistics failed this criterion, we reported the evidence provided by the data in terms of the posterior distribution (posterior mean, HDI and evidence ratios) but refrained from making definitive conclusions.

#### 2.8.2 Power analysis and sample size rationale

To estimate the power of this study, we used the posterior distribution from the model estimated in our previous study (Boayue et al., 2020) to simulate random datasets, fitting back the analytical model and estimating the probability of finding an effect where 95% of the posterior density is in the expected direction. We were most interested in the coefficients for AE, BV, AE × BV and the interaction between Group and Block (reflecting the effect of real vs. sham brain stimulation protocols). As a practical upper limit, due to time-constraints in the career track of the first author and because of the ongoing pandemic posing restrictions on our lab-work, we set a maximum sample-size of $N_{max}=50$ per group resulting in a total of 100 participants with valid datasets.

For the effects of BV, AE and their interaction, we created random datasets by drawing samples of increasing sample-sizes from the posterior distribution from the previous study and creating thought-probe responses using the model predictions (see https://osf.io/ctmbw/for the R-script). For each of these random datasets, we fit back the original model (with non-informative prior distributions as in our previous study) and calculated the posterior estimate of the regression coefficients. Next, for each random dataset, we calculated whether we were successful in obtaining an estimate for which the posterior distribution was largely (95%) in the expected direction (positive for BV, negative for AE and positive for the interaction). Finally, we collapsed across random datasets of the same sample size to calculate the proportion of successful fits, which gave us an estimate of statistical power for the decision-rule that 95% of the posterior-distribution was in the expected direction. The advantage of this approach is that, instead of using only a point-estimate for the effect of interest, we automatically incorporate the uncertainty from the posterior distribution of the previous study into the power-estimate.

**Fig. 3:**
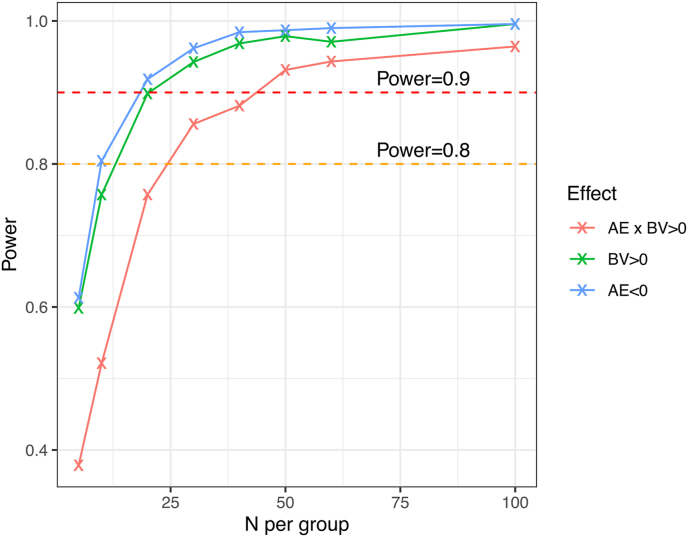
Power-curves for the effects of BV, AE and BV × AE calculated based on the full posterior distribution.

We calculated the power that way using sample-sizes from the set N∈{5,10,20,30,40,50,60,100} and simulated between 700 and 7000 random datasets. The reason for the varying number of simulated datasets is computational runtime which was excessive for the larger sample-sizes. The resulting power-curves are shown in Fig. 3. The power curves for both AE and BV cross the threshold of 90% power quite early (around N = 20) while the interaction effects require larger samples (between 40 and 50 participants per group) to reach this threshold.

For the effect of HD-tDCS on mind wandering, we used a similar approach as above, except that we simulated datasets that had varying effect-sizes as indicated by different standardized regression coefficients for the Block × Group effect. The reason for that was that we wanted to get a fuller picture of which effect-sizes could still be resolved with a certain power given intermediate sample sizes. We therefore randomly generated datasets of both varying sample sizes and varying effect-sizes, re-fit the models and calculated the percentage of “successful” studies where 95% of the posterior distribution was negative (the expected effect was in the negative direction, i.e., we expected HD-tDCS to reduce self-reported mind wandering, see Boayue et al., 2020).

Unfortunately, the computational power required to calculate the power-curves with sufficient precision was prohibitive. We therefore ran the power-calculations for a grid of sample-sizes and effect-sizes for one week on a server with 80 cores and used a parametric model fit to provide an approximation to the actual power-curves. The simulation-based power-calculations are coded in the script https://osf.io/ctmbw/. We chose an asymptotic exponential growth model to calculate estimated power $\hat{\beta }$ based on the standardized regression coefficient b and the sample size per group N as

$$\hat{\beta }\left(b,N\right)=1-exp\left(-\left[m_{0}+m_{1}b+m_{2}b^{2}\right]N\right).$$

**Fig. 4:**
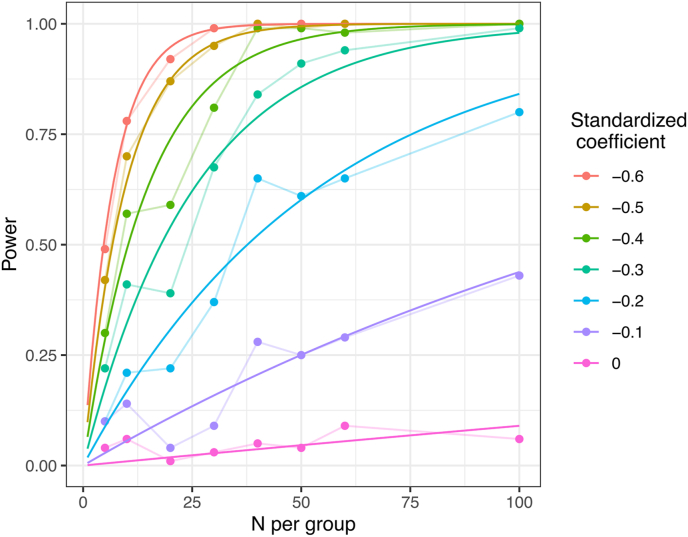
Fit of the asymptotic exponential growth model to directly calculated power-curves.

Consequently, the rate of increase of the power curve with sample size N is modulated by the effect-size b where this modulation takes the form of a quadratic linear regression. Fitting this model to the data results in estimates for the coefficients $m_{0}$, $m_{1}$ and $m_{2}$ which allows the approximation of power for any effect-size and sample-size. The fit of this model to the simulated power-curves is illustrated in Fig. 4. The power-analysis based on this approximation was deemed to result in an acceptable fit (Fig. 4) and is available in the R-script at https://osf.io/w73xj/.

**Fig. 5:**
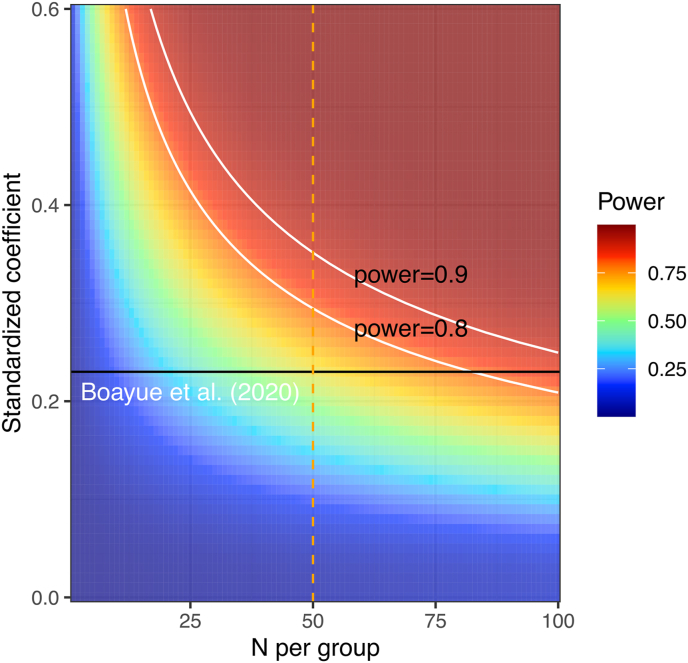
Approximate power of the effect of HD-tDCS on mind wandering as a function of sample-size and effect-size. The solid, black line marks the previously measured effect-size, the dashed line is our maximum sample size of N = 50 per group. The white isolines show regions of 80 and 90 percent power.

Next, based on this approximate power-analysis, we calculated the power-surface as a function of sample- and effect-size (Fig. 5). It appears that approximately N = 80 subjects per group would be required to achieve 80% power for the same effect size we measured previously (the intersection of the black and the first white line).

**Fig. 6:**
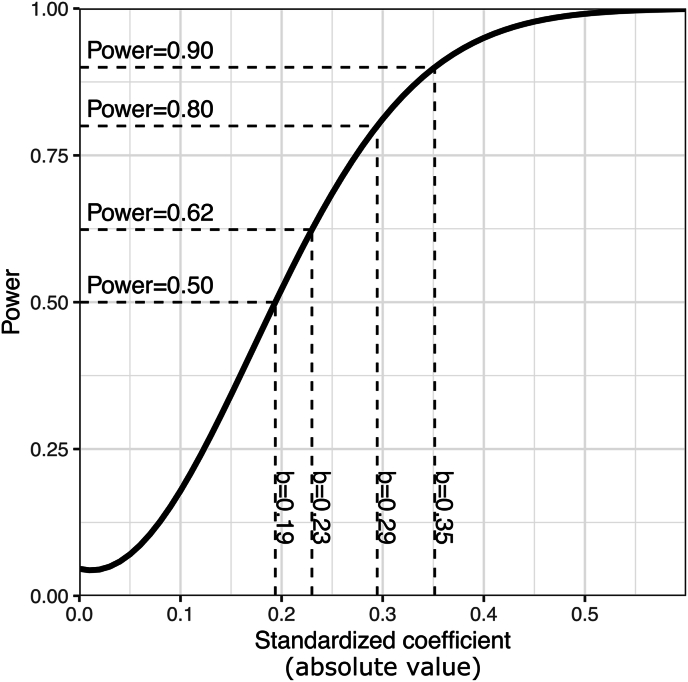
Power of the effect of HD-tDCS on mind wandering for N = 50.

Since we decided not to exceed a maximum of $N_{max}=50$ subjects per group for practical reasons, we investigated power for N=50 in more detail by plotting power as a function of standardized regression coefficient (Fig. 6). For a real effect-size that is as large as measured in our previous study (b=−.23), we have approximately 62% power to detect it. We have still an even chance to detect effect-sizes of b<−.19. To achieve 80 and 90% power, the real effect-size would have to be b=−.29 and b=−.35, respectively. As the sample-size required to achieve 80% power in all our target analyses exceeds our pre-specified $N_{max}=50$ per group, we decide to collect data until 100 valid datasets (N = 50 per group) have been accumulated.

### 2.9 Exploratory analyses

In addition to providing a direct replication of Boayue et al. (2020) as formulated in the four pre-registered hypotheses specified above, we extended the study protocol in a more exploratory manner to get a better understanding of the neural mechanisms underlying these effects. These extensions, i.e., an additional experimental block featuring oddball-stimuli and the recording of pupillometry and EEG-data, did not interfere with the exact replication protocol. We consider our hypotheses regarding these measures as exploratory and not part of the stricter pre-registration and therefore specified them more loosely.

#### 2.9.1 Prolonged effects

Modulatory effects on cortical excitability induced by tDCS are assumed to outlast the duration of the actual stimulation. We therefore hypothesized that the effect of real HD-tDCS on MW would persist even after stimulation has ended. Hence, we hypothesized that the effect would be unchanged in the final (offline) block. Concretely, when extending the pre-registered statistical model described above by including the third, offline block as an additional level in the Block variable, we hypothesized that the Group × Block interaction will be similar for the stimulation- and the offline-block.

#### 2.9.2 Robustness of the effects to changes of the time-window preceding probes

In order to test whether our results are sensitive to the choice of the pre-probe temporal window, we repeated the main ordered-probit regression analysis using a range of choices for this parameter. Specifically, we compared the regression coefficients from models fitted to data extracted using 10, 15, 20 and 25 trials before each probe.

#### 2.9.3 Meta-analytic integration with Boayue et al. (2020)

To draw empirical-based conclusions about our hypotheses, we wanted to quantify the total amount of evidence for the presence or absence of the effect of HD-tDCS on MW propensity provided by both the original study (Boayue et al., 2020) and our current study. A suitable approach to this is to combine the two datasets to calculate an estimate of the effect-size that maximizes precision (Braver et al., 2014). A prerequisite for that analytical strategy is that the two studies are homogenous so that they can be assumed to measure the same effect. The target-effect in our study was a (negative) coefficient in a Bayesian hierarchical ordinal regression model and no standard measure of heterogeneity exists for that situation.

We therefore pursued the following strategy to ensure homogeneity of the studies. First, we compared the baseline sessions of the two studies to ensure that any incidental differences between the two studies (see section 2.7) did not result in significant differences in behavior. Specifically, we contrasted self-reported MW, AE and BV between the two sessions using side-by-side comparisons and, more formally, by implementing an ordinal regression model which included interaction terms with a study-level covariate coding from which study a datapoint originated. In case the 95% HDI of any of these coefficients excludes zero, we can conclude that the effects between the studies are heterogenous. It is important that these blocks were comparable as our main outcome was a within-subject effect between baseline and stimulation block and therefore, it could have been strongly affected by performance in the baseline block. Comparability between the two baseline blocks allowed us to proceed with merging the previous dataset of N = 60 from Boayue et., al (2020) with the current data, reaching a total sample size of N = 160 (N = 80 per HD-tDCS condition). We repeated the ordered probit regression analysis detailed above on this full sample, adding group-level covariates coding for the study and possible interactions with the other variables.

#### 2.9.4 Pupillometry

As described above, we extracted tonic and phasic pupil dynamics for single trials using both traditional methods (i.e., using average pupil diameter (PD) in pre-stimulus and post-stimulus time-windows) and an experimental tonic- and phasic estimation algorithm (Mittner, 2020). We tested the hypothesis that switches between on-task and MW periods are accompanied by transient increases in tonic (baseline) pupil size. Given the fast pace of our experimental task, we assumed that the traditional method would be ineffective in extracting valid baseline measures due to a build-up of the pupillary responses to stimuli. Hence, we hypothesized that the observed relationship would be stronger for the estimation algorithm compared to traditional averaging across the pre-stimulus window. In addition, we hypothesized that tap-associated pupil responses would be larger when being on-task or in periods of high AE, both indicating periods of task engagement that should evoke stronger stimulus-related pupillary responses. Similar as for the baseline PD, we quantified the phasic responses using both a traditional averaging and our estimation algorithm and assumed a stronger relationship for the latter.

#### 2.9.5 EEG

We were interested in comparing MMN amplitudes from the offline block between the two experimental groups. Since we expected to find increased MW propensity in the offline block following sham HD-tDCS, this group was also expected to show reduced neural responses to oddball stimuli, supporting the *perceptual decoupling* account of MW (Braboszcz and Delorme, 2011; Groot et al., 2021; Smallwood and Schooler, 2015). Conversely, real HD-tDCS was expected to result in enhanced MMN amplitudes, indicating a more alert task-focus state, and more efficient detection of unpredictable external events.

Fluctuations in occipital alpha power were also compared between blocks (baseline vs. offline) and groups (real vs. sham HD-tDCS). We hypothesized that MW reports would be accompanied by stronger occipital alpha power (Braboszcz and Delorme, 2011; Macdonald et al., 2011; O'Connell et al., 2009), and therefore, we also anticipated enhanced alpha activity in the offline vs. the baseline block (representing the neural correlate of the well-established *time-on-task effect* of MW frequency). With respect to our hypothesis regarding HD-tDCS effects, we also expected a more modest increase in alpha power from the baseline to the offline block in the real HD-tDCS group, compared to the sham group. Similarly, the sham HD-tDCS group was expected to show more prominent alpha activity in the offline block, being indicative of more frequent attentional lapses and MW reports.

Furthermore, as midfrontal theta power (MFT) is thought to reflect neural activity associated with executive control (Cavanagh and Frank, 2014), we expected MFT and the generation of random sequences to be correlated throughout the task on a single trial level. Next, we wanted to compare changes in MFT between blocks and HD-tDCS conditions. Even though response randomness (quantified by AE) did not change over the task and was not influenced by HD-tDCS in our previous study (Boayue et al., 2020), we expected MFT to be increased in the real vs. sham stimulation group in the offline block. This hypothesis was based on empirical evidence regarding the involvement of executive processes in the onset and maintenance of MW episodes (Christoff et al., 2016; Smallwood et al., 2012). In particular, both the EFf and EFu accounts of MW posit that less executive resources are directed to the external task while being engaged in MW, and therefore, response-locked midfrontal theta should be weaker following sham HD-tDCS, characterized by more frequent MW. Thus, we argued that midfrontal theta may be a more sensitive marker for task-oriented executive resources than AE itself and could provide important insights into the MW-EF relationship.

Finally, we wanted to calculate mean MFT across the 25 trials preceding each thought probe. These values were used as outcome variable to assess their relationship to time-on-task (Block: baseline vs. offline), mental state (State: MW vs. on-task), HD-tDCS (Group: real vs. sham) and their interactions. We hypothesized that the negative association between MW and AE (see pre-registered hypotheses, section 2.8.1) would be accompanied by a negative MW-MFT relationship. In addition, the negative MW-MFT association was anticipated to be enhanced in the offline block in participants receiving real HD-tDCS, which would provide support for the EFf view of MW (i.e., the “gatekeeper” function of the executive system to prevent intrusive MW episodes becomes more prominent following DLPFC stimulation).

## 3 Results

### 3.1 Demographics

Our sample consisted of 100 participants (54 females), aged 18–36 years (mean age = 24.58). Due to our inclusion criteria, there were no excluded datasets. In total eight participants had to be replaced. Seven of these were replaced immediately during data collection, and one participant had to be replaced after data inspection. Out of the seven participants who were replaced during data collection, two participants failed to provide complete dataset due to lack of responses to target stimuli, four participants were accidently tested with the incorrect protocol, or the incorrect protocol sequence was run, and one participant was familiar with the study protocol and hypothesis from an earlier study. Additionally, four participants had problematic data (almost twice as many taps as required, only responding “completely-on task” on all probes, pressing too few keys, or only pressing the same key). Out of those we decided that only one participant could be excluded based on pre-registered criteria (failure to provide a complete dataset due to not responding to target stimuli). The full dataset, as well as all eight excluded subjects are available on OSF (https://osf.io/cv24f/).

### 3.2 Effect of HD-tDCS stimulation on mind wandering

#### 3.2.1 Pre-registered hypotheses

According to our pre-registration, we used an ordinal regression model that included AE, BV, their interaction, Trial (probe number), Block (baseline vs. stimulation), Group (sham vs. real stimulation) and their interaction to analyze the responses to the thought-probes. The model yielded an adequate fit to the data, R^2^_bayes_ = 0.35.

There was no difference between the two groups in the baseline block (*b* = −0.05 [−0.35, 0.23], ER- = 1.54) in terms of self-reported MW. We confirmed our hypotheses that BV would be increased prior to self-reports of mind wandering when compared to on-task periods (*b* = 0.19 [0.14, 0.24], ER_+_ = ∞) and that the utilization of executive resources measured by AE would be reduced prior to mind wandering (*b* = −0.07 [−0.11, −0.03], ER- = 362.64). However, we did not find any evidence for the expected positive interaction between BV and AE (*b* = 0.00 [−0.03, 0.04], ER_+_ = 1.36). Finally, and most importantly, we did not find any effect on the propensity to mind wander in the real relative to the sham stimulation group during the stimulation block of the experiment (*b* = 0.00 [−0.19, 0.19], ER- = 1.01).

Note: The reported R^2^ is based on the “bayes_R2” function in the brms package in R, where predictors are treated as continuous variables.

#### 3.2.2 Time on task, prolonged stimulation, and other task effects

Furthermore, as expected, we found strong evidence for the well-known time-on-task effect on mind wandering, i.e., increased mind wandering as task duration increases. This effect manifested both within each individual block and, on a longer timescale, across blocks. Compared to baseline, we found increased mind wandering in the stimulation block (*b* = 0.27 [0.13, 0.40], ER_+_ = 1332.33) and we also found a positive effect of Trial within-blocks (*b* = 0.81 [0.68, 0.95] ER_+_ = ∞).

To investigate potential prolonged stimulation effects (“offline” effects), we refit the model with inclusion of the offline block. We observed almost no changes to the regression coefficients reported above when adding the offline block to the analysis (BV; *b* = 0.21 [0.17, 0.26], ER_+_ = ∞, AE; *b* = −0.07 [−0.1, −0.03], ER- = 665.67, BV × AE; *b* = 0.00 [−0.03, 0.04], ER_+_ = 1.41). There was no change in self-reported MW propensity between the sham and the real stimulation group in the offline block when compared to baseline (*b* = 0.00 [−0.22, 0.21], ER- = 1.05), indicating that there was no evidence for an effect of HD-tDCS on MW propensity in our experiment. The time on task effect estimated by this model was slightly stronger (stimulation block, *b* = 0.24 [0.09, 0.38], ER_+_ = 306.69, offline block, *b* = 0.24 [0.10, 0.40], ER_+_ = 265.67, probe number *b* = 0.91 [0.78, 1.04], ER_+_ = ∞).

#### 3.2.3 Robustness to changes in time-window preceding probes

**Fig. 7:**
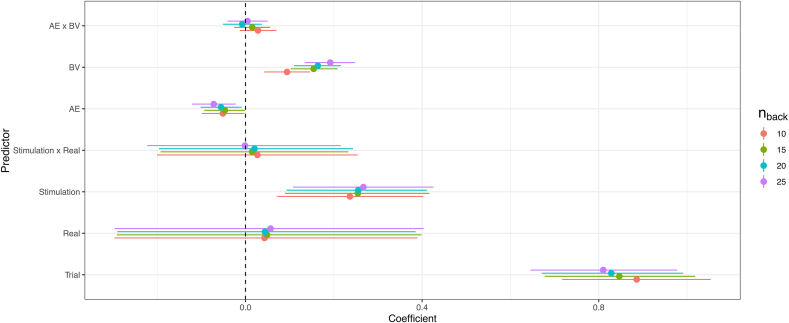
Coefficient estimates using different numbers of trials (n_back_) before each probe.

Next, we rerun the above model (including only baseline and stimulation blocks) for varying values of the n_back_ parameter that indicates how many trials before each probe were used to calculate AE and BV. Adjusting this parameter did not change the qualitative effects of BV and AE on MW propensity. However, consistent with Boayue et al. (2020), the magnitude of the effects for AE and BV was increasing with the number of trials included (Fig. 7). Specifically, the effect of increased MW with increased BV was weakest for n_back_ = 10 (*b* = 0.0932 [0.0422, 0.142]) and strongest for n_back_ = 25 (*b* = 0.191 [0.135, 0.247]) and the effect of decreased MW with increased AE was weakest for n_back_ = 10 (*b* = −0.051 [−0.0982, −0.0039]) and strongest for n_back_ = 25 (*b* = −0.072 [−0.122, −0.0236]).

#### 3.2.4 Meta-analytic integration with Boayue et al. (2020)

**Fig. 8:**
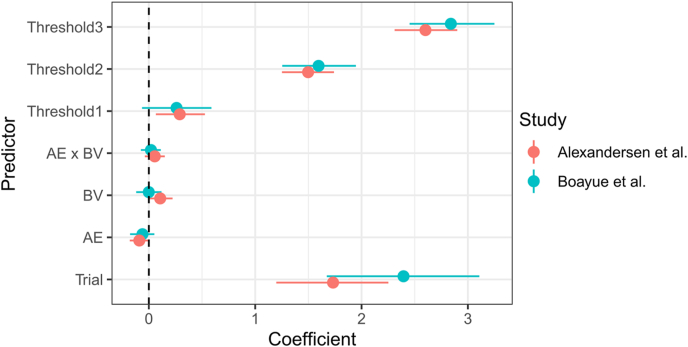
Comparison of the coefficients in Boayue et al. (2020) and the present study in the baseline block using independent regression models. “Threshold1-3” represent the thresholds distinguishing between different responses on the Likert scale estimated in the hierarchical ordered probit model.

Before integrating our dataset with the original dataset collected in Boayue et al. (2020), we compared the baseline blocks of both studies to test whether there were major differences between the two studies. For that purpose, we fit a hierarchical ordered probit model to the dataset combining the baseline blocks from both studies and included BV, AE, Trial, and Study as variables. The Study variable was allowed to interact with all other variables to determine whether any of the observed relationships between MW and the predictor variables were different between the two studies. We found that none of the 95% HDIs of the coefficients representing interactions with the study variable excluded zero (Study × BV: *b* = −0.11 [−0.28, 0.05], Study × AE: *b* = 0.04 [−0.10, 0.18], Study × Trial: *b* = 0.53 [−0.37, 1.42], Study × AE × BV: *b* = −0.03 [−0.17, 0.10]) and we therefore conclude, per our pre-registered criterion, that the two baseline blocks were comparable. However, when comparing the coefficient estimates from independent models for the two studies side-by-side (Fig. 8), we noted some discrepancies (most notably a reduced coefficient for BV, and an increased time-on-task effect in Boayue et al., 2020; BV_Alexandersen_: *b* = 0.11 [−0.002, 0.22], BV_Boayue_: *b* = −0.003 [−0.12, 0.11], Trial_Alexandersen_: *b* = 1.73 [1.21, 2.26], Trial_Boayue_: *b* = 2.39 [1.65, 3.14]) though these were not strong enough to trigger our pre-registered criteria.

Therefore, we combined the two datasets to reach the full sample of N = 160 and re-ran the original model while allowing all predictors besides block and group to interact with Study. Based on that analysis, the 95% HDI of the effect of HD-tDCS on MW did not exclude zero (*b* = −0.08 [−0.22, 0.06]) indicating that the stimulation effect on MW propensity disappeared when looking at all of the available data combined. The combined estimates for the remaining predictors were in line with our previous results (BV: *b* = 0.20 [0.14, 0.26], AE: *b* = −0.07 [−0.13, −0.02], Trial: *b* = 0.87 [0.71, 1.03], Block: *b* = 0.25 [0.12, 0.37]). Regarding the interactions of these predictors with the Study variable, the 95% HDI of the coefficient for BV excluded zero (Study x× BV: *b* = −0.10 [−0.18, −0.02]) while the remaining coefficients did not (Study × AE: *b* = −0.05 [−0.13, −0.04], Study × Trial: *b* = 0.03 [−0.23, 0.28], Study × BV × AE: *b* = 0.03 [−0.04, 0.10]).

### 3.3 Blinding

**Fig. 9:**
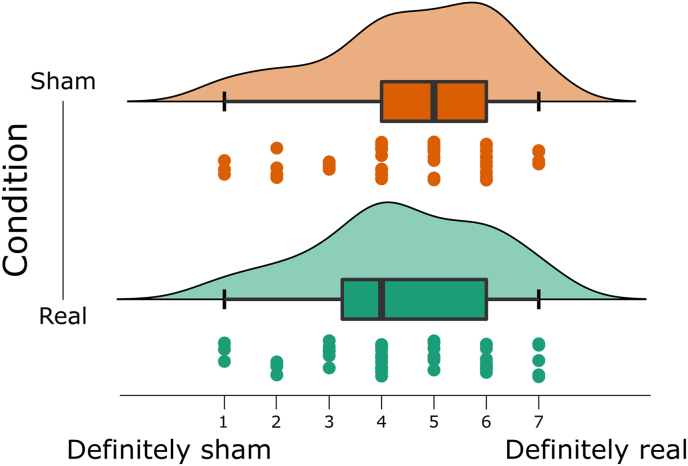
Distribution of self-reports to the blinding questionnaire between the two groups.

To assess whether participants were successfully blinded with respect to HD-tDCS condition, we compared the two groups in their scores on a 7-point Likert scale asking whether they believed they received real stimulation or not. We performed a two-sided Bayesian Mann-Whitney test which supported the null hypothesis (BF_01_ = 4.16), providing evidence that participants were successfully blinded to real versus sham stimulation. The distributions depicted in Fig. 9 even show a slight tendency for the opposite effect.

### 3.4 EEG

#### 3.4.1 Mismatch negativity

**Fig. 10:**
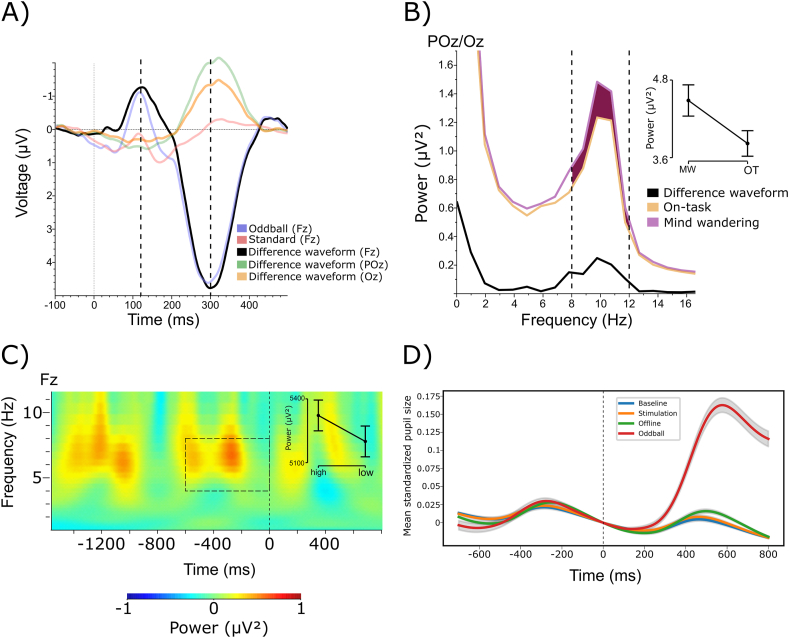
Preliminary checks for MMN, occipital alpha power, midfrontal theta power and evoked pupil dilation. A) Mismatch negativity waveform in the expected time-window (120–300 ms, highlighted with dashed vertical lines) on electrode Fz referenced to the signal from the mastoids (P9/P10), which switches polarity on electrode POz and Oz. B) Increased occipital alpha power (indicated with dashed vertical lines) preceding MW thought probes compared to probes where participants reported to be on-task. C) Enhanced MFT in the pre-stimulus time-window ([-500 ms, 0] ms) in the expected frequency range (4–8 Hz) for high entropy trials compared to low entropy trials (time-frequency area of interest highlighted with the dashed rectangle). D) Evoked pupil response (grand average of standardized pupil size across [-700, 800] ms window relative to metronome onset) to the standard metronome tones in each of the three blocks and to the oddball stimuli in the offline block.

**Fig. 11:**
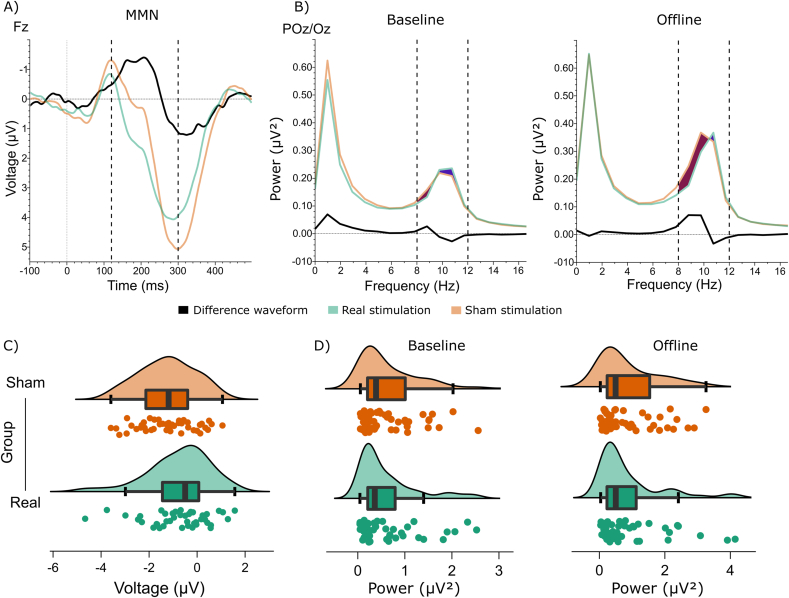
A) MMN waveforms for the sham and real HD-tDCS groups, along with the sham-real difference waveform (black). B) Power spectra for the sham and real HD-tDCS groups, along with the sham-real difference trace. Vertical dashed lines represent the time window (A) and the frequency range (B) of interest. C) Individual MMN amplitudes and D) posterior alpha power values for the two groups with corresponding boxplots and density plots.

As anticipated, we observed a reliable mismatch negativity to the oddball stimuli. Specifically, we found an oddball-standard difference waveform of negative polarity peaking between 120 and 300 ms at electrode Fz, and reversed polarity at posterior-inferior electrodes POz and Oz (Fig. 10A). To investigate whether HD-tDCS influenced the MMN amplitude in the offline block, we performed a one-tailed independent samples Bayesian *t*-test to assess the directional hypothesis that the MMN standard-oddball difference waveform at electrode Fz would be larger (more negative) following real stimulation. This assumed effect would indicate enhanced reactivity of the auditory system to salient, unexpected events, accompanying the anticipated reduction in MW propensity in the real HD-tDCS group. However, in line with the null-effect of HD-tDCS on probe responses, this analysis also favored the null hypothesis (BF_01_ = 12.048, Fig. 11A and C).

#### 3.4.2 Occipital alpha power

To assess whether occipital alpha (8–12 Hz) power was indicative of MW, we performed a one-tailed Bayesian Wilcoxon signed-rank test (due to excess skewness and kurtosis in the data) to test whether alpha power was enhanced during MW vs. OT periods. The result supported the hypothesis that occipital alpha power preceding MW probes was stronger compared to OT probes (BF_10_ = 7.61, Fig. 10B). As the next step, we included data from all segments, not only those preceding thought probes to increase statistical power, and performed a Bayesian repeated-measures ANOVA on occipital alpha power with Block (baseline vs. offline) as within-subject, and Group (real vs. sham HD-tDCS) as between-subject factor. We found strong evidence for the main effect of Block, indicating a time-on-task effect of enhanced alpha power in the final experimental block (BF_incl_ = 5582.29). Crucially, the main effect of Group (BF_incl_ = 0.38) and the interaction term (BF_incl_ = 0.32) both favored the null hypothesis (Fig. 11B and D).

#### 3.4.3 Frontal midline theta power

To assess whether MFT (4–8 Hz) power reflected the recruitment of executive control, we tested the directional hypothesis that MFT was stronger for high AE vs. low AE trials. For this purpose, we extracted MFT power in the [−500,0] ms time-window preceding each motor response and performed a one-tailed Bayesian Wilcoxon signed-rank test (due to excessive skewness in the data). Our results support the anticipated effect (BF_10_ = 6.81, Fig. 10C).

**Fig. 12:**
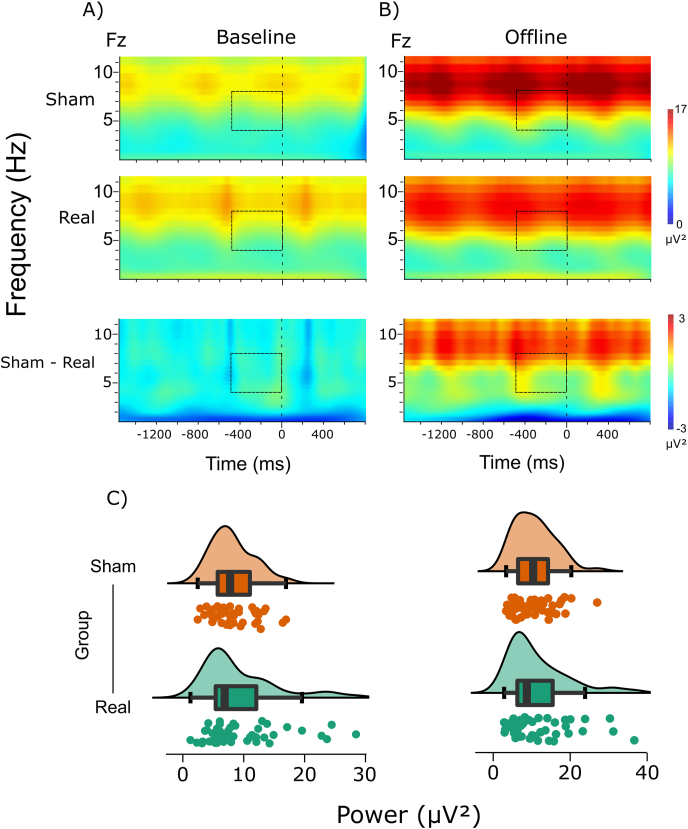
MFT burst preceding motor responses (at stimulus onset, 0 ms) for A) baseline and B) offline blocks, for the two groups, and for the sham-real HD-tDCS group difference (dashed rectangles represent the time-frequency area of interest). C) Individual MFT power data for the two groups and blocks (left: baseline block, right: offline block), with corresponding boxplots and density curves.

Next, we conducted a Bayesian repeated-measures ANOVA with Block (baseline vs. offline) as within-subject and Group (real vs. sham) as between-subject factors, including data from all trials. Our results reveal strong evidence for a main effect of Block (BF_incl_ = 2524.00) indicating that MFT was increased in the offline relative to the baseline block. Importantly, even though we expected enhanced MFT power in the offline block following real stimulation, for both the main effect of Group and the Block × Group interaction, statistical evidence supported the null hypothesis (BF_incl_ = 0.34 and BF_incl_ = 0.21, respectively) see Fig. 12.

As a final step, we extracted mean MFT power for the 25 trials preceding thought-probes, and conducted a Bayesian repeated-measures ANOVA with Block (baseline vs. offline) and State (on-task vs. MW) as within-subject, and Group (real vs. sham) as between-subject factors. Contrary to our hypotheses, we only found evidence for the main effect of Block (BF_incl_ = 3.72 × 10^6^), while other BFs for the main effects and all interactions supported the null hypothesis (State: BF_incl_ = 0.18, Group: BF_incl_ = 0.50, Block × State: BF_incl_ = 0.20, Block × Group: BF_incl_ = 0.21, State × Group: BF_incl_ = 0.20, Block × State × Group: BF_incl_ = 0.29).

### 3.5 Pupillometry

To further investigate the neurophysiological processes involved in MW, we assessed changes in pupil size during the FT-RSGT, by extracting stimulus-locked evoked pupil responses centered around the metronome sounds in all three experimental blocks (see Fig. 10D).

#### 3.5.1 Missing data

We removed all trials in which more than 20% of the pupillometric signal was missing in the time-window used for extracting the single-trial measure. This resulted, on average, in the loss of 19.0% of the trials (SD = 14.2%). When calculating per-probe pupillometric measures, we used the same window of preceding 25 trials as we did for the behavioral data. We excluded probes where more than 10 of the 25 trials before each probe were excluded because of missing data, resulting in the loss of 81 out of a total of 2920 trials.

#### 3.5.2 Comparison of the traditional and novel algorithm

From the raw pupillometric time-series, we extracted per-trial measures of tonic and phasic activity using a traditional and our novel method (see section 2.6.1). The average correlation between traditional and novel estimates of tonic fluctuations in pupil size was very high (r=0.99,SD=0.07). In contrast, the correlation between the two methods for extracting pupillary responses to task events (phasic activity) was much smaller (r=0.22,SD=0.25). This reflects the way in which the novel algorithm enforces the tonic estimate to be a lower envelope of the pupillometric signal, therefore affecting mostly the transient phasic signal.

#### 3.5.3 Pupil responses and mind wandering

In order to investigate the relationship between tonic and phasic pupillary dynamics and MW, we conducted two separate hierarchical ordered probit regression models (one for the traditional and one for the novel estimation algorithm) with MW as the dependent variable. As predictors, we included tonic and phasic pupil (and their interaction) as well as trial number, experimental block (baseline, stimulation, offline) and their interaction. The overall Bayesian $R^{2}$ for the two models was $R^{2}=0.29$ (traditional) and $R^{2}=0.30$ (novel).

Contrary to our expectations, larger tonic pupil size was associated with decreased MW (b=−0.08[−0.14,−0.01], traditional estimate: b=−0.05[−0.11,0.02]) and larger phasic pupil responses were associated with increased MW (b=0.14[0.01,0.27], traditional: b=0.05[−0.24,0.33]), even though both effects were only evident in the model based on features extracted using the novel algorithm. In addition, we observed a tonic × phasic interaction effect (b=0.16[0.001,0.32], traditional: b=0.02[−0.39,0.43]), suggesting that the relationship between phasic pupil responses and MW was dependent on changes in tonic pupil size. Specifically, for high tonic levels (mean + 2 × SD), the relationship between phasic pupil-reponse and MW was strongly positive (b=0.31[0.06,0.55]), for mean tonic levels weakly positive (b=0.1[−0.02,0.12]), and zero for low tonic levels (mean – 2 × SD, b=−0.11[−0.34,0.12]).

As before, the effect of trial number on MW was positive (b=0.07[0.04,0.10], traditional: b=0.07[0.04,0.10]). In addition, MW was increased during the stimulation block (b=0.37[0.14,0.60], traditional: b=0.37[0.14,0.60]) but not during the final offline block (b=0.09[−0.17,0.36], traditional: b=0.08[−0.18,0.33]). The effect of trial number on MW was reduced during the stimulation block (b=−0.03[−0.06,−0.0002], traditional: b=−0.03[−0.06,−0.0007]) but unaffected during the offline block (b=0.02[−0.02,0.07], traditional: b=0.03[−0.01,0.07]).

#### 3.5.4 Pupil response and HD-tDCS

**Fig. 13:**
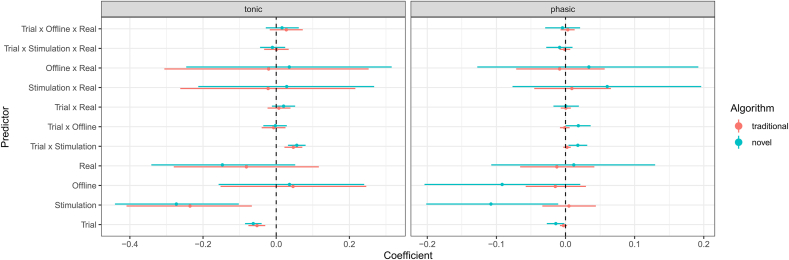
Predictors and coefficients from the traditional and novel algorithm from the four Bayesian hierarchical linear regression models, with tonic (left) and phasic (right) pupil responses as dependent variables.

To test whether the pupillary measures were affected by real vs. sham stimulation, we conducted separate Bayesian hierarchical linear regression models with tonic and phasic pupil as dependent variables. We used Trial, Block, Group, and their interactions as predictors. The resulting coefficient estimates for the four models are presented in Fig. 13. While the results for the traditional and novel analysis for the tonic pupillary levels are very similar (as expected based on their high correlation), the phasic response as extracted from the two algorithms show very different signatures.

With increasing trial number, tonic pupil dilation was reduced (b=−0.063 [−0.085,−0.040], traditional: b=−0.052 [−0.076,−0.030]). In addition, during the longer stimulation block, tonic pupil was reduced (b=−0.27 [−0.44,−0.10], traditional: b=−0.236 [−0.41,−0.067]) but not during the final offline block that included oddball stimuli (b=0.036[−0.16,0.241], traditional: b=0.046[−0.152,0.246]). There was no difference between the sham and the real groups with regards to tonic pupil dilation (b=−0.15[−0.34,0.052], traditional: b=−0.081[−0.28,0.12]) and the difference in tonic levels between baseline and stimulation block was not affected by real stimulation either (b=0.029[−0.21,0.27], traditional: b=−0.022[−0.26,0.22]). The same was true for the offline block: The change in tonic pupil size from baseline to the last offline block was not affected by real stimulation (b=0.036[−0.25,0.32], traditional: b=−0.008[−0.071,0.057]). During the stimulation block, the effect of trial number was reduced (b=0.056[0.032,0.080], traditional: b=0.047[0.023,0.071]) but that effect was not influenced by whether the stimulation was sham or real (b=−0.010[−0.044,0.025], traditional: b=0.001[−0.033,0.034]). During the offline block, the effect of trial number was unchanged relative to baseline (b=−0.002[−0.035,0.029], traditional: b=−0.007[−0.040,0.025]) and unaffected by sham vs. real stimulation (b=0.016[−0.028,0.062], traditional: b=0.028[−0.018,0.073]).

With respect to the phasic pupillary response, the results from the traditional algorithm are inconclusive because none of the coefficients’ HDIs excluded zero (Fig. 13). With respect to the results from the novel algorithm, the phasic pupillary response decreased with increasing trial number (b=−0.014[−0.027,−0.002]) and also during the stimulation block compared to baseline (b=−0.11[−0.20,−0.011]) but not in the offline block (b=−0.092[−0.20,0.021]). However, the negative relationship between phasic pupil responses and trial number was reduced during the stimulation block (b=0.018[0.004,0.031]) and during the offline block (b=0.019[0.000,0.036]). Real stimulation did not affect the phasic response (b=0.012[−0.11,0.130]) and the decrease in phasic pupil response from baseline to the stimulation block (b=0.060[−0.077,0.20]) and from baseline to offline block (b=0.034[−0.13,0.19]) were also unaffected by real stimulation. Finally, real stimulation did not change the effect of trial number in either baseline block (b=0.000[−0.017,0.019]), stimulation block (b=−0.009[−0.028,0.010]), or offline block (b=−0.004[−0.029,0.021]).

#### 3.5.5 Pupil responses and approximate entropy

Finally, we investigated whether the tonic and phasic pupil dynamics were related to the AE of the sequence calculated over a 25-trial window backwards in time. For that purpose, we calculated four separate Bayesian hierarchical linear models with AE as dependent variable. Each model had a single predictor (tonic and phasic pupil, each measured using the traditional and the novel approach) and included random intercepts and slopes at the subject level. Tonic pupil was not related to AE (b=−0.012[−0.069,0.042], traditional: b=−0.006[−0.063,0.052]). The direction of the relationship between AE and phasic pupil responses was as expected, indicating increased phasic pupil responses during periods of high AE, but the 95% HDI did not exclude zero (b=0.064[−0.030,0.16], traditional: b=−0.016[−0.241,0.204]).

## 4 Discussion

The main goal of the current study was to replicate the effect that MW propensity can be reduced by HD-tDCS of the left DLPFC reported by Boayue et al. (2020) using a high-powered, pre-registered protocol. However, we found that HD-tDCS did not reduce or otherwise affect the propensity to MW in our study. There was no evidence for a difference in MW propensity between the real vs. sham groups during the stimulation block, nor a prolonged effect of real HD-tDCS in the offline block. To include all available evidence for the effectiveness of HD-tDCS on manipulating MW propensity, we performed a meta-analytic integration of the present study and Boayue et al. (2020), reaching a total sample size of N = 160. Unsurprisingly given the results of our high-powered study, the combined analysis also failed to show an effect of HD-tDCS on the propensity to MW. We therefore conclude that the earlier finding by Boayue et al. (2020) was most likely a statistical fluke and that it is not possible to modulate the propensity of MW using HD-tDCS with our proposed protocol.

The sobering experience from the current study highlights the necessity of pre-registration of studies utilizing HD-tDCS - and non-invasive brain stimulation methods more broadly - to affect cognitive functions. We believe that the study by Boayue et al. (2020) was methodologically advanced relative to most other studies in the field: They used a within-subject repeated-measures design (comparison against individual baselines), a highly sensitive task and analysis method, a state-of-the-art stimulation protocol which ensured a properly double-blinded application of the stimulation, and a substantial sample-size. In addition, they established analytical choices for the HD-tDCS dataset based on pilot experiments that did not include brain stimulation to reduce the risk of confirmatory bias and to limit researchers’ degrees of freedom. Furthermore, even though they did not pre-register their final analysis pipeline, the analyst was blinded against the coding of the group variable (i.e., the analyst completed the analysis using arbitrary labels for sham vs. real stimulation groups). Despite these cautious measures that are far from the standard in the field, the authors detected an effect of the stimulation on MW propensity and only the careful replication with pre-registered methods reported here was able to convincingly show that this effect is not actually real. We therefore argue that the evidential value of studies reporting a positive effect of tDCS on MW, unless validated by a rigorous, pre-registered replication, is very low.

In part, this discouraging conclusion may be accounted for by a glaring need for improved NIBS methods compared to standard methods applied in most studies to date. First, even though the growing number of studies applying HD-tDCS protocols is a step in the right direction to increase focality of the stimulation, between-subject variability in brain anatomy may counteract that positive development, i.e., a more focal stimulation method applied at a similar scalp location may target different underlying cortical areas in different participants. A possible way forward could be to embrace prospective electric field modelling for individual participants (Evans et al., 2020; Rasmussen et al., 2021) which would allow to individually place stimulation electrodes to target the same underlying brain areas. Second, all studies in the field so far (including our own) rely on introspective thought-probes to quantify MW. The inherently subjective nature of this measure, which may be affected by a wide variety of nuisance variables, makes it a “moving target” for any interventional method, including NIBS. One way forward may be to rely more on objective behavioral or neurophysiological measures such to eliminate the need for thought probes altogether (Groot et al., 2021; Kucyi et al., 2017).

Our proposed behavioral task, the FT-RSGT, seems to be well-suited for the purpose of providing more objective measures of MW as it provides two relevant behavioral indices, AE and BV, at high temporal resolution and can be readily combined with neural measures. Our study confirmed the expected relationship between these indices and MW, where BV was increased, and AE reduced during periods of MW. The FT-RSGT also captures well-known task markers such as time on task effects, both between and within blocks, providing solid evidence of the robustness of the FT-RSGT how the high temporal nature of the task gives reliable behavioral manifestations of MW. Additionally, we could not replicate the predicted AE × BV interaction. We are therefore forced to disconfirm our previous speculation that this interaction may represent different manifestations of MW (Boayue et al., 2020; Mittner et al., 2016).

We extended the study with pupillometric measures in order to investigate the relationships between dynamic changes in phasic and tonic pupil size and MW that have been hypothesized to be related to MW (Mittner et al., 2016). Specifically, we hypothesized that switches between on-task and MW periods would be accompanied by transient increases in tonic pupil size (baseline PD), and that phasic pupil responses would be stronger during periods of on-task or high AE. Contrary to our expectations, we found that MW was associated with decreases in tonic pupil size. This suggests that as participants disengaged from the finger-tapping task, tonic norepinephrine was reduced, whereas during periods of on-task focus, tonic levels increased. Previous studies regarding the relationship between tonic pupil size and MW have reported inconsistent results (Grandchamp et al., 2014; Groot et al., 2021; Jubera-García et al., 2020; Konishi et al., 2017) indicating that this relationship is possibly dependent on specific task effects. The current results add support to the findings from a recent fMRI study employing the same task (FT-RSGT), which also found a negative relationship between tonic pupil and MW (even though their HDI did not exclude zero and the effect was therefore considered inconclusive; Groot et al., 2022).

Surprisingly, we observed increased phasic pupillary responses to task events associated with periods of MW, contradicting previous findings and the established theory that periods of task disengagement coincide with perceptual decoupling (Smallwood et al., 2012; Unsworth and Robison, 2018). One possible explanation is that the stimuli themselves are not particularly task-relevant in the FT-RSGT (only their timing is). As a consequence, during periods of strong task-focus, perhaps the processing of the stimulus was inhibited in order to suppress distractions from the internal calculation of the entropy. Our analysis furthermore revealed that the dependence between phasic pupil and MW was dependent on levels of tonic pupil size, showing that the positive relationship between phasic responses and MW was strongest when tonic pupil size was high, but diminished when tonic pupil size was low. This might indicate that the low-tonic mode was associated with reduced alertness (Unsworth and Robison, 2018), thereby leveling out any relationship between MW and phasic responses (which are also expected to be reduced during low tonic activity).

Furthermore, we explored whether changes in tonic and phasic pupil size would be related to improved task performance in terms of AE. The results show that although phasic responses were positively related to AE, the HDIs for neither tonic nor phasic predictors excluded zero and therefore these effects are considered inconclusive and require targeted follow-up studies to be established.

Finally, we also sought to investigate whether our novel algorithm would result in more valid pupil features, given the fast-paced nature of our task and the expected build-up in pupil dilation that complicates reliable estimation of both tonic and phasic changes in pupil size. While the correlation between tonic (baseline) pupil size extracted using traditional and novel methods was high, phasic pupil responses extracted using the novel estimation algorithm differed from those using the traditional averaging method. In addition, most of the reported effects, for example the relationship between phasic responses and MW, were only evident when using pupillary features estimated with the novel algorithm. We therefore conclude that our novel approach was able to account for the sluggish response of the pupil to events and to isolate temporally precise effects in our fast-paced task.

By analyzing EEG data, our primary aim was to find support for the expected effect of HD-tDCS on the neural level, by clarifying whether modulation of MW by real stimulation is accompanied by changes in the MMN, posterior alpha power, MFT, or any combination thereof. While we were only able to collect data from 8 electrodes, we found multiple well-known neurophysiological markers associated with MW, executive control and sensory prediction errors. In accordance with the perceptual decoupling theory (Braboszcz and Delorme, 2011), we hypothesized that we would find reduced MMN amplitude during periods of higher MW propensity following sham HD-tDCS. However, in line with the behavioral null-effect, we did not see any group difference in the MMN in the offline block. This result does not preclude that MMN is not a sensitive neurophysiological marker of MW, but in the context of non-invasive brain stimulation, more potent protocols are warranted to clarify if changes in MW are associated with reduced neural sensitivity to unexpected and salient sensory events. The null-effect is not only interesting from the MW perspective, but also because frontal tDCS could theoretically influence MMN more directly via changing excitability in its frontal generators (Chen et al., 2014; Weigl et al., 2016). However, even with our HD-tDCS protocol, we did not observe any influence of HD-tDCS on the MMN.

Given that occipital alpha power is an established marker for MW (Hawkins et al., 2015), we expected and found increased alpha-band activity preceding thought probes where participants reported to be engaged in MW when compared to being on-task. Additionally, we found that occipital alpha power showed a clear time-on-task effect, being larger in the offline vs. baseline block. In line with our finding on the null-effect of HD-tDCS on MW, we found no difference in occipital alpha power between the real and sham stimulation groups in the crucial offline block.

Additionally, we found the anticipated effect of MFT being enhanced preceding high AE responses compared to low AE responses. This provides further evidence that MFT is a marker for increased task performance in the FT-RSGT, and the relationship between MFT and AE indicates that MFT can be regarded as a neural index of implementing executive control (Cavanagh and Frank, 2014). Again, in line with the behavioral results, HD-tDCS did not influence MFT, as there was no noticeable difference between the real and sham groups. However, while these results are in line with HD-tDCS not influencing self-reported task focus, it is quite surprising that real stimulation above the left DLPFC did not modulate MFT or AE, since this region has been closely associated with executive control (Royall et al., 2002). We also expected to observe reduced MFT as task duration increases, as participants would get more tired and shift to MW more frequently. Instead, we found a strong effect in that MFT was increased in the offline block compared to both baseline and stimulation blocks. Possible explanations could be that as task duration increased, participants become more tired, and a compensatory increase in MFT power could reflect efforts in an attempt to stay awake and focused on the task (Klimesch, 1999, Strijkstra et al., 2003) While we observed an increase in MFT as task duration increased, this did not manifest in self-reports of being focused on the task, suggesting that merely recruiting MFT to “fight” inattentiveness was not sufficient to perform well.

Alternatively, stronger MFT power in the offline block could have been due to the oddball stimuli. We observed stronger increase in MW in the stimulation block compared to the offline block (relative to baseline), therefore it is possible that the oddballs might have increased alertness (in line with the phasic pupil effect), and this task-unspecific alerting (tones were not related to the primary AE-focused task) could have improved the recruitment of executive resources, manifesting in stronger MFT.

In sum, we did not observe any effect of HD-tDCS on MW or any of our behavioral or neural measures. However, we found stable and robust behavioral markers for MW reflected in the FT-RSGT. We verified the association between occipital alpha power and MW, as well as the relationship between MFT and executive control. Finally, we provided novel insights into the link between tonic/phasic pupil responses and MW, indicating that the direction of the association is vulnerable to task-effects, and might be influenced by changes in general arousal.

## Declaration of competing interest

The authors declare that they have no known competing financial interests or personal relationships that could have appeared to influence the work reported in this paper.

## Footnotes

1 The details described in the following paragraphs were not specified in the pre-registration document submitted to OSF before data-collection. However, none of the files containing pupillometric or EEG data were opened prior to writing up the manuscript.
